# High-quality draft genome of Gammaproteobacterial SUP05 cluster from non-buoyant hydrothermal plumes of ultraslow spreading Gakkel Ridge (Central Arctic Ocean)

**DOI:** 10.1128/mra.00121-24

**Published:** 2024-03-08

**Authors:** Stefano Scilipoti, Massimiliano Molari

**Affiliations:** 1 HGF MPG Joint Research Group for Deep Sea Ecology and Technology, Max Planck Institute for Marine Microbiology, Bremen, Germany; DOE Joint Genome Institute, USA

**Keywords:** hydrothermal plume, Central Arctic Ocean, deep sea, SUP05, metagenome-assembled genomes, *Thioglobaceae*

## Abstract

Hydrothermal plumes are an important yet understudied component of deep-sea microbial ecosystems. We report metagenome-assembled genomes (MAGs) of three Bacteria belonging to the Gammaproteobacterial SUP05 cluster (family *Thioglobaceae*), assembled from the metagenomes of two non-buoyant hydrothermal plumes in the ultraslow spreading Gakkel Ridge.

## ANNOUNCEMENT

Deep-sea hydrothermal plumes are generated when hot, chemically reduced hydrothermal fluids are emitted from seafloor vents and mixed with cold and oxic deep-sea water (1). Despite their recognized important role in ocean biogeochemistry (2), little is known about the taxonomic and functional diversity of microbes that inhabit the hydrothermal plume (3). We present three metagenome-assembled genomes (MAGs) members of the SUP05 cluster (*Gammaproteobacteria*), widely spread in the oceans and dominant in hydrothermal plumes (4).

Seawater samples were collected at two hydrothermal plumes of the Gakkel Ridge in the Central Arctic Ocean (5, 6). Microbial cells were sampled with *in situ* pumps (WTS-LV04; McLane) equipped with polycarbonate filters (0.2 µm pore size; Millipore), which were frozen in liquid nitrogen and stored at −80°C. DNA was extracted using the DNeasy PowerWater Kit (Qiagen), following the manufacturer’s instructions. For each station, paired-end libraries were prepared with the TruSeq DNA PCR-Free Sample Prep Kit (Illumina), and sequencing of libraries was performed on a MiSeq 2500 instrument (Illumina; 2 × 300 cycles) using v3 sequencing chemistry with 3 million reads per library (CeBiTec laboratory). Unless indicated, the following bioinformatics tools were used with default parameters. Reads had adapters and contaminants removed with the bbduk tool in the BBMap suite (http://jgi.doe.gov/data-and-tools/bb-tools/; k = 27; mink = 12) and were trimmed with Trimmomatic (slidingwindow:4:20; minlen:100; v0.35) (7). Forward and reverse reads were assembled using metaSPAdes within the suite SPAdes (“--meta,” v. 3.9.0) (8). Reads were mapped and indexed using bwa (v0.7.12) (9) and samtools (v1.5)(10), and the scaffolds were binned with CONCOCT (v1.1.0) (11). Bins were refined using Anvi’o interactive interface (v6.1) (12) after an anvi’o contig database was built to calculate k-mer frequencies, identify open reading frame using Prodigal (v.2.6.3) (13), single-copy genes using HMMER (v.3.2.1) (14), and to classify the bins based on single copy gene taxonomy of GTDB (15) using DIAMOND (v 0.9.14) (16). 16S rRNA genes were extracted with RNAmmer (v.1.2) (17). Refined bins were re-assembled using BBmap (99% similarity) and SPAdes (default parameters), removing contigs smaller than 1 kb. Completeness and contaminations of the final bins were evaluated using CheckM (v1.0.18) (18). Three bins were classified to the family of *Thioglobaceae*, with a completeness of 94%–95% and very low or null contamination (Table 1). 16S rRNA gene similarity assigned the three bins to the Thioglobus genus of the SUP05 clade, and the similarity between the bins was >98.3%. Average nucleotide identity was calculated using JSpeciesWS (v. 4.1.1) (19) for the three bins. ANIb values suggest that one bin (SUP05_GR1) is a different species from the other two bins (SUP05_GR2a and SUP05_GR2b), the latter bins sharing a 97.55% ANIb value, higher than the accepted threshold of 94-95% similarity used for species classification (20). Based on 16S rRNA gene similarity and ANIb, we propose that the three bins belong to two new species and that SUP05_GR2a and SUP05_GR2b are two strains of the same species.

**TABLE 1:** Statistic for draft genomes of SUP05 and information for sampling location

	SUP05_GR1	SUP05_GR2a	SUP05_GR2b
No. scaffolds	48	10	10
Total length (bp)	1081737	1260588	1209183
GC content (%)	39.42	40.73	40.84
Completeness	94.93	95.09	94.53
Contamination	4.31	0.28	0
Strain Heterogeneity**^*a*^**	100	100	0
Scaffold N50	35495	283305	218876
No. protein-coding genes	1152	1335	1277
No. rrna	3	3	3
No. trna	28	35	35
No. tmrna	1	1	1
Longest scaffold	70396	386034	235476
Coding density	0.9388	0.9310	0.9345
Mean scaffold length	22536	126058	120918
Quality**^*b*^**	medium	high	high
Accession number	GCA_963932455.1	GCA_963932435.1	GCA_963932445.1
Station label	PS101-181	PS101-188	PS86-40
Sampling time	03.10.16	05.10.16	17.07.14
Sampling Lat.	86.951	86.949	82.918
Sampling Long.	55.74	55.678	−6.377
Water depth (m)	2635	2635	3183

^*a*^ The strain heterogeneity indicates the proportion of the contamination that appears to be from the same or similar strains.

^*b*^ Based on Bowers et al. (21).

## Data Availability

The clipped reads used for the assembly and MAGs were deposited under the study accession number PRJEB36324. The run accessions for the reads are ERR3830158, ERR3830157, ERR3830159, and the accession numbers for MAGs are GCA_963932455.1, GCA_963932435.1, and GCA_963932445.1

## References

[1] German CR, Seyfried WE Jr. 2014. Hydrothermal processes, p 191–233. In Holland HD, Turekian KK (ed), Treatise on geochemistry, 2nd ed. Elsevier, Oxford, UK.

[2] Elderfield H, Schultz A. 1996. Mid-ocean ridge hydrothermal fluxes and the chemical composition of the ocean. Annu Rev Earth Planet Sci 24:191–224. doi:10.1146/annurev.earth.24.1.191

[3] Dick GJ, Anantharaman K, Baker BJ, Li M, Reed DC, Sheik CS. 2013. The microbiology of deep-sea hydrothermal vent plumes: ecological and biogeographic linkages to seafloor and water column habitats. Front Microbiol 4:124. doi:10.3389/fmicb.2013.00124 23720658 PMC3659317

[4] Dick GJ. 2019. The Microbiomes of deep-sea hydrothermal vents: distributed globally, shaped locally. Nat Rev Microbiol 17:271–283. doi:10.1038/s41579-019-0160-2 30867583

[5] Boetius A. 2015. The expedition PS86 of the research vessel POLARSTERN to the Arctic ocean in 2014. doi:10.2312/BzPM_0685_2015

[6] Boetius A, Purser A. 2017. The expedition PS101 of the research vessel POLARSTERN to the Arctic ocean in 2016. doi:10.2312/BzPM_0706_2017

[7] Bolger AM, Lohse M, Usadel B. 2014. Trimmomatic: a flexible trimmer for Illumina sequence data. Bioinformatics 30:2114–2120. doi:10.1093/bioinformatics/btu170 24695404 PMC4103590

[8] Nurk S, Meleshko D, Korobeynikov A, Pevzner PA. 2017. metaSPAdes: a new versatile metagenomic assembler. Genome Res 27:824–834. doi:10.1101/gr.213959.116 28298430 PMC5411777

[9] Li H, Durbin R. 2009. Fast and accurate short read alignment with Burrows – Wheeler transform. Bioinformatics 25:1754–1760. doi:10.1093/bioinformatics/btp324 19451168 PMC2705234

[10] Li Heng, Handsaker B, Wysoker A, Fennell T, Ruan J, Homer N, Marth G, Abecasis G, Durbin R, 1000 Genome Project Data Processing Subgroup. 2009. The sequence alignment/map format and SAMtools. Bioinformatics 25:2078–2079. doi:10.1093/bioinformatics/btp352 19505943 PMC2723002

[11] Alneberg J, Bjarnason BS, de Bruijn I, Schirmer M, Quick J, Ijaz UZ, Lahti L, Loman NJ, Andersson AF, Quince C. 2014. Binning metagenomic contigs by coverage and composition. Nat Methods 11:1144–1146. doi:10.1038/nmeth.3103 25218180

[12] Eren AM, Esen ÖC, Quince C, Vineis JH, Morrison HG, Sogin ML, Delmont TO. 2015. Anvi’o: an advanced analysis and visualization platform for ‘omics data. PeerJ 3:e1319. doi:10.7717/peerj.1319 26500826 PMC4614810

[13] Hyatt D, Chen G-L, Locascio PF, Land ML, Larimer FW, Hauser LJ. 2010. Prodigal: prokaryotic gene recognition and translation initiation site identification. BMC Bioinformatics 11:119. doi:10.1186/1471-2105-11-119 20211023 PMC2848648

[14] Eddy SR. 2011. Accelerated profile HMM searches. PLoS Comput Biol 7:e1002195. doi:10.1371/journal.pcbi.1002195 22039361 PMC3197634

[15] Parks DH, Chuvochina M, Waite DW, Rinke C, Skarshewski A, Chaumeil P-A, Hugenholtz P. 2018. A standardized bacterial taxonomy based on genome phylogeny substantially revises the tree of life. Nat Biotechnol 36:996–1004. doi:10.1038/nbt.4229 30148503

[16] Buchfink B, Xie C, Huson DH. 2015. Fast and sensitive protein alignment using DIAMOND. Nat Methods 12:59–60. doi:10.1038/nmeth.3176 25402007

[17] Lagesen K, Hallin P, Rødland EA, Staerfeldt HH, Rognes T, Ussery DW. 2007. RNAmmer: consistent and rapid annotation of ribosomal RNA genes. Nucleic Acids Res 35:3100–3108. doi:10.1093/nar/gkm160 17452365 PMC1888812

[18] Parks DH, Imelfort M, Skennerton CT, Hugenholtz P, Tyson GW. 2015. CheckM: assessing the quality of microbial genomes recovered from isolates, single cells, and metagenomes. Genome Res 25:1043–1055. doi:10.1101/gr.186072.114 25977477 PMC4484387

[19] Richter M, Rosselló-Móra R, Oliver Glöckner F, Peplies J. 2016. JSpeciesWS: a web server for prokaryotic species circumscription based on pairwise genome comparison. Bioinformatics 32:929–931. doi:10.1093/bioinformatics/btv681 26576653 PMC5939971

[20] Richter M, Rosselló-Móra R. 2009. Shifting the genomic gold standard for the prokaryotic species definition. Proc Natl Acad Sci USA 106:19126–19131. doi:10.1073/pnas.0906412106 19855009 PMC2776425

[21] Bowers RM, Kyrpides NC, Stepanauskas R, Harmon-Smith M, Doud D, Reddy TBK, Schulz F, Jarett J, Rivers AR, Eloe-Fadrosh EA, et al.. 2017. Minimum information about a single amplified genome (MISAG) and a metagenome-assembled genome (MIMAG) of bacteria and archaea. Nat Biotechnol 35:725–731. doi:10.1038/nbt.3893 28787424 PMC6436528

